# Metal Nanocluster-Based Biosensors for DNA Detection

**DOI:** 10.3390/bios15020072

**Published:** 2025-01-25

**Authors:** Ran He, Sheng Wang, Feiye Ju, Zhao Huang, Yuan Gao, Jing Zhang, Nongyue He, Libo Nie

**Affiliations:** Hunan Key Laboratory of Biomedical Nanomaterials and Devices, College of Life Sciences and Chemistry, Hunan University of Technology, Zhuzhou 412007, China; m22077700021@stu.hut.edu.cn (R.H.); m23077700008@stu.hut.edu.cn (S.W.); m22077700026@stu.hut.edu.cn (F.J.); huang1020@hut.edu.cn (Z.H.); gaoyuan@hut.edu.cn (Y.G.); zhang_jing@hut.edu.cn (J.Z.)

**Keywords:** biosensor, DNA probe, metal nanocluster, fluorescence, DNA hybridization

## Abstract

The early detection of genetic diseases is a critical need in modern medicine, underscoring the importance of developing deoxyribonucleic acid (DNA) biosensors. In recent years, metal nanoclusters (MNCs) have demonstrated significant potential as biosensors for DNA detection due to their ultra-small size, excellent photostability, bright photoluminescence, low toxicity and other outstanding properties. This review firstly discusses the characteristics of MNCs, which are effective in the early diagnosis of DNA diseases. Subsequently, different synthesis methods of MNCs are introduced. In the following section, DNA sensors based on different types of MNCs and their respective detection mechanisms are discussed in detail. Finally, the opportunities and challenges faced by DNA sensors based on MNCs are analyzed.

## 1. Introduction

After decades of development, nanomaterials are still a research area of wide concern [1]. Metal at the nanoscale can be categorized into metal nanoparticles and metal nanoclusters (MNCs) based on size regimes [2,3]. Atomically accurate nanoclusters (NCs) have different physical and chemical properties from nanoparticles (NPs) and are attracting more and more attention in different application fields, which further expands the vision of nanomaterial science. MNCs usually consist of tens to hundreds of atoms, with a core size of less than 2 nm, which is close to the Fermi wavelength of electrons, placing them between individual metal atoms and metal NPs [4,5,6,7,8]. The pronounced quantum confinement effect in MNCs leads to the division of continuous energy states into discrete ones. Their ultra-small size and discrete energy levels give rise to strong photoluminescence, redox capabilities, HOMO-LOMO transitions, large Stokes’ shift, molecular magnetism, and other unique physical and chemical properties [5,9,10,11,12]. These features make MNCs promising in various applications like cancer treatment [13], antimicrobial therapy [14], imaging and sensors [15]. Among the features, their fluorescence property is particularly attractive, providing a pathway for high-performance sensors, imaging agents, photosensitizers and more. Sensors based on MNCs have been widely used in biomedical detection.

MNC-based sensors can be used to detect biological molecules such as deoxyribonucleic acids (DNAs), microRNAs, and proteins. As important biological macromolecules, DNAs are not only associated with the biological inheritance and the transmission of genetic information [16,17,18], but also with the occurrence of many diseases [19,20]. For example, single KRAS G12D-activated mutations often result in colorectal carcinoma [21]; HPV-16 DNA is associated with the heightened risk of cervical cancer [22]; CYFRA21-1 serves as an important marker for detecting lung cancers [23]. Therefore, the early and accurate detection of DNAs is of great significance in healthcare. Although traditional detection methods such as DNA polymerase chain reaction (PCR) [24,25], enzyme-linked immunosorbent assay [26], and microarray analysis [27] are known for their high reliability, here are some disadvantages linked to them, such as high cost, complex operation, and high requirements for sample quality. DNA biosensors possess advantages such as ease of use, low cost, miniaturization, high sensitivity, and effectiveness [28,29]. New DNA detection methods include electrochemical sensing [30,31], surface plasma resonance, surface-enhanced Raman scattering, fluorescence and colorimetric sensing and so forth [32,33,34]. High sensitivity, selectivity, and rapid responsiveness are important parameters for sensors. The characteristics of MNCs offer significant advantages in optimizing these parameters [10,35,36,37]. Compared with traditional biosensors (such as quantum dots and organic dyes), MNCs possess characteristics such as strong light absorption and emission properties, good photostability, and biocompatibility. Moreover, by precisely controlling the size, shape, and composition of MNCs, the optical, electrical, and magnetic properties of MNCs can be regulated, thereby enhancing their interaction with DNA molecules and improving signal transduction, which greatly enhances the detection sensitivity and accuracy of biosensors [38]. Therefore, MNC-based sensors are a good option for the early and accurate detection of DNA.

This review focuses on MNCs that can be used in the early detection of DNA diseases. Firstly, various synthesis methods for MNCs, such as chemical reduction, ligand exchange, chemical etching, and other methods are introduced. In the following section, DNA sensors based on gold nanocluters (AuNCs), silver nanocluters (AgNCs), copper nanocluters (CuNCs) and bimetallic nanocluters, as well as corresponding detection mechanisms, are discussed in detail. Finally, the challenges in sensor development and potential solutions are addressed. This review aims to serve as a valuable resource for the design of future MNC-based DNA detection sensors and to inspire further research and innovation in MNC-based luminescent sensors.

## 2. Synthesis of Metal Nanoclusters

**Figure 1:**
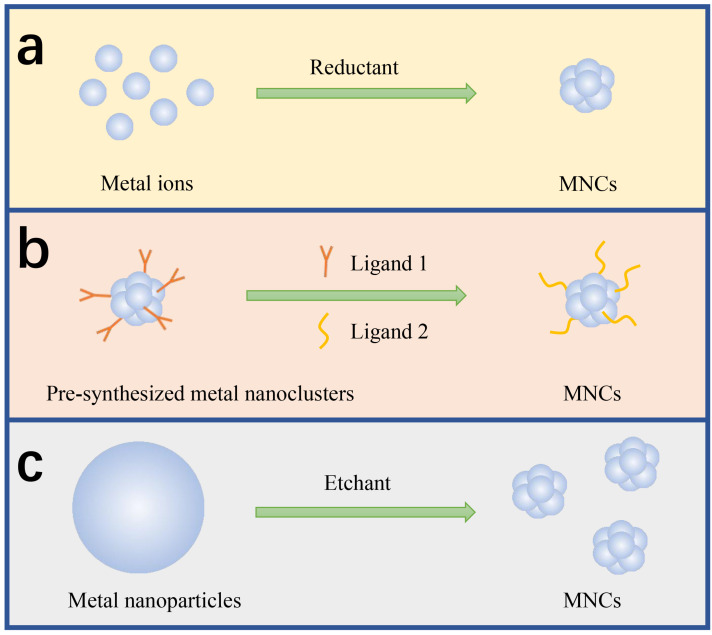
Schematic illustration of synthetic methods for MNCs: (**a**) chemical reduction; (**b**) ligand exchange; (**c**) chemical etching.

The great interest in synthesizing MNCs stems from their remarkable and diverse applications across various fields of modern science [9,39,40]. Therefore, numerous chemical and physical methods have been developed for the synthesis of MNCs with precise atomic compositions [41]. Up to now, atomically precise MNCs can be easily prepared through different synthesis methods [42,43]. As shown in Table 1, those methods include chemical reduction (Figure 1a), ligand exchange (Figure 1b), chemical etching (Figure 1c), and so on. For the early detection of DNA, MNCs with proteins or DNAs serving as ligands are typically synthesized.

### 2.1. Chemical Reduction

As the most used method, chemical reduction is a “bottom-up” synthetic strategy. In a typical process, metal ions are reduced to lower-valent-state atoms in solution, followed by clustering them to form MNCs [44]. Reducing agents include Sodium Borohydride (NaBH_4_), ascorbic acid, glutathione (GSH), etc. The reducing agent NaBH_4_ can enhance the fluorescence quantum yield; the fluorescence emission wavelength of MNCs coated with the reducing agent GSH may shift; the reducing agent ascorbic acid can maintain the photophysical properties of MNCs. The speed of the reduction process and the strength of the reducing agent are crucial to the success of the reaction. The method is straightforward and rapid, and it can be conducted under mild conditions.

In the chemical reduction synthesis of MNCs, the most commonly used reducing agent is NaBH_4_, which has mild reduction conditions. Zeng et al. reported the synthesis of the most stable medium-sized Au_64_(S−c-C_6_H_11_) with a molecular weight of less than 20 kDa using NaBH_4_ as a reducing agent [45]. The synthetic process consists of two parts. In the first part, Au(III) is converted to Au(I) via cyclohexanethiol, forming Au(I)S−c-C_6_H_11_ complexes, and the strong reducing agent NaBH_4_ is added to further reduce Au(I) to Au(0). The coarse Au_m_(S−c-C_6_H_11_)_n_ clusters can be thereby obtained. In the second part, the crude product is dissolved in toluene and cyclohexanethiol, and it is gently stirred at 90 °C to obtain single-size Au_64_(S−c-C_6_H_11_)_32_ clusters. Chen et al. also used NaBH_4_ as a reducing agent to develop a simple, gentle and rapid method for synthesizing well-dispersed AuAgNCs [46]. Firstly, HAuCl_4_ was added to ultrapure water and mixed with AgNO_3_ to form a flocculent precipitation. Next, GSH and NaBH_4_ was added to the mixture in icy water. Finally, NaOH was added to adjust the pH to 7.5. The GSH@AuAgNCs could be formed within 15 min at room temperature. Nasirian and coworkers reported that DNA-stabilized AgNCs could be prepared by mixing DNA, AgNO_3_, NaBH_4_ at an optimal molar ratio of 1:18:18 [47]. The synthetic reaction took 6 h in the dark environment.

Ascorbic acid, as a reducing agent, is often used to synthesize CuNCs. For example, a facile method to prepare water-soluble fluorescent CuNCs templated by nucleosides was reported [48]. In the synthesis, ascorbic acid was used as the reducing agent and nucleosides as stabilizers. During the formation of CuNCs, Cu(II) in copper nitrate were reduced to copper atoms by ascorbic acid, and then agglomerated to form CuNCs at 80 °C. At the same time, nucleotides comprised CuNCs to improve stability. Under neutral conditions, Tiwari et al., using adenine and thymine as templates, highly stable and highly fluorescent CuNCs with a very unique negative CD peak of about 350 nm were synthesized for the first time [49]. During the synthesis process, CuNCs exhibiting maximum fluorescence were synthesized by utilizing an optimal concentration of the reducing agent ascorbate at 2 mM and the optimal combination of DNA/Cu^2+^/ascorbate at a ratio of 1:1000:400.

In addition to the above two, GSH has also been reported as a reducing agent in the chemical reduction synthesis of MNCs, and it can also act as a ligand. In 2018, Katla et al. reported a method for the rapid synthesis of AuNCs with excellent photothermal activity within 2 h without low-temperature conditions and agitation [50]. L-GSH was added to the HAuCl_4_ solution, reducing Au(III) to Au(I). To this mixture, NaBH_4_ was added to further reduce Au(I) to Au(0). Finally, methanol was added to precipitate AuNCs, which were obtained by centrifugation and by removing the supernatants. Yan et al. successfully synthesized stable CuNCs by utilizing GSH as both a reducing agent and protecting agent [51]. The turbid liquid mixture of CuCl_2_ and GSH was clarified with NaOH and then stirred at 80 °C for 24 h to yield CuNCs, which can be further utilized after being washed with isopropyl alcohol multiple times.

### 2.2. Ligand Exchange

The ligand exchange method allows selective replacement of the surface ligands of MNCs to produce new MNCs [52,53]. This method can impact the core size, geometry, solubility, and photoelectric properties of MNCs significantly. The ligand exchange reaction can control the size of MNCs through a fast and direct process.

Liu and coworkers reported that Au_144_(SCH_2_Ph)_60_NCs were obtained from polydispersed Au*_n_* (SG)*_m_*NCs in the presence of excess phenyl methanethiol (H−SCH_2_PH) ligands via multiple “ligand exchange” processes [54]. Toluene, ethanol and H−SCH_2_PH were added to Au*_n_* (SG)*_m_*NCs, and Au*_n_* (SCH_2_PH)_*m*_NCs were prepared by the exchange of ligand (SG${)}_{m}^{n-}$ and (SCH_2_PH${)}_{m}^{n-}$. Shichibu and coworkers reported a large-scale synthesis of thiolate-capped Au_25_ clusters via a ligand exchange reaction with phosphine-stabilized Au_11_ clusters [55]. Meanwhile, they found that thermodynamically stable Au_25_(SG)_18_ can be selectively obtained on the sub 100 mg scale under controlled conditions. Various ligands have been used to synthesize AgNCs, including DNA [56], thiolates [57], polymers [58], peptides [59], and proteins [60]. Chang et al. conducted a ligand exchange reaction, replacing the ligands of Ag_21_[S_2_P(OiPr)_2_]_12_ with NH_4_[Se_2_P(OEt)_2_] to produce Ag_21_[Se_2_P(OEt)_2_]_12_ clusters [61]. Bootharajuet et al. reported the interconversion between Ag_25_(SPhMe_2_)_18_NCs and Ag_44_(SPhtF)_30_NCs. Exchanging the ligands of non-hollow Ag_25_(SPhMe_2_)_18_ with excess HSPhtF ligands yielded hollow Ag_44_(SPhtF)_30_NCs [62].

Compared with AuNCs and AgNCs, the ligand exchange method is relatively less used in the synthesis of CuNCs, but there are some related reports. Nguyen et al. reported the synthesis of novel Cu_29_NCs based on the cluster growth initiated by the ligand exchange reaction [63]. In the synthetic process, the ligand exchange reaction produces CuCl_2_ monomers, which are then captured by Cu_25_NCs to generate Cu_29_NCs. Cu_29_NCs are the largest copper superatoms known to date.

### 2.3. Chemical Etching

Chemical etching is achieved by etching large NPs into small NCs, which is a “top-down” synthetic strategy [64,65]. MNCs can be obtained by etching metal nanoparticles using different etching agents.

At present, the etch agents reported include GSH, H_2_O_2_, cysteamine hydrochloride, and ammonia. Then, a series of descriptions are given on the synthesis of MNCs using these etchers. Le Guével’s group converted silver nanoparticles to non-bleaching and highly photostable AgNCs via GSH as the ligand etchant [66]. By changing the reaction time with the etchant (one day, three days, and eight days), the samples were washed several times with acetone, and AgNCs with red, blue and yellow fluorescence were obtained, respectively. Chen and coworkers reported a facile, simple and rapid chemical etching method for preparing AuNCs capped with luminol at room temperature [67]. Firstly, AuNCs were synthesized by a mixed solution of HAuCl_4_ and luminol, and then the GSH etchant was added to obtain green-fluorescent AuNCs.

Liu et al. found that etching AgNCs with H_2_O_2_ led to the formation of smaller clusters, increasing the fluorescence intensity of GSH-AgNCs [68]. In the study by Shen et al., bovine serum albumin stabilized gold nanoparticles (AuNPs) were obtained by a typical reduction method [69]. Then, a solution comprising AuNPs was mixed with the etchant, cysteamine hydrochloride, at a volume ratio of 1:3, to etch AuNPs to form AuNCs. Deng’s group reported that the conversion of non-luminescent copper NPs to luminescent CuNCs was achieved by using ammonia as the etchant [70]. The etching process using ammonia gas is rapid, taking 15 min, and yields CuNCs with strong green fluorescence.

### 2.4. Other Synthetic Methods

Microwave-assisted and green synthesis technologies are becoming increasingly important in the field of MNCs synthesis, not only by helping to improve synthesis efficiency and reduce costs but also by having a lesser impact on the environment, thus facilitating the achievement of more sustainable and cost-effective synthesis methods. For example, Saleh and colleagues developed a microwave-assisted synthesis method, which is fast and environmentally friendly. In this method, the pepsin molecule is used as a stabilizer and reductant. The mixture of copper nitrate, pepsin and sodium hydroxide is irradiated in a microwave oven for 30 min, and then the microwave-assisted solution is dialyzed overnight in ultrapure water to obtain pepsin CuNCs. There are some synthesis methods that use ultrasound or microwave, and the whole process does not use any additional reducing agent [71]. Sadhu and coworkers reported a facile, microwave method to fabricate blue-fluorescent CuNCs from the extract of *Bacopa monnieri* [72]. Li et al. used poly metacrylicacid, sodium saltas on the surface ligand to synthesize water-soluble fluorescent AgNCs by microwave radiation [73]. This method is fast, simple, and highly repeatable. For the first time, Naaz’s team has synthesized AgNCs with photoluminescence through fine tuning of sunlight and ultrasound [74]. A more convenient method was also mentioned, and Zhang et al. reported the synthesis of natural silk fibroin-stabilized CuNCs by a one-pot method, which has excellent water solubility and pH responsiveness [75]. The sensor based on it has a fast response and a wide detection range.

## 3. MNCs for DNA Detection

This section discusses different MNC-based sensors for DNA detection. DNA, a biological macromolecule with good biocompatibility and water solubility, serves as the most commonly used template for MNCs [76]. DNA-templated MNCs have shown great potential in biochemical sensing, including DNA detection [77,78,79]. Besides DNA, proteins are also effective templates for MNCs used in DNA detection [80,81]. This section introduces four types of MNCs, namely AuNCs, AgNCs, CuNCs, and alloy NCs, as DNA sensors.

### 3.1. AgNCs for DNA Detection

AgNCs have been at the forefront of research due to their ease of synthesis, good photostability, and the lower cost compared to their gold counterparts. AgNCs are mainly used in biological sensing [82,83]. Among all the AgNCs, DNA-templated AgNCs exhibit the most excellent physical and chemical properties [4], in terms of quantum yield, photostability, redox, emission tunability and very high specific surface product properties [84,85]. The properties of MNCs are largely influenced by the design of DNA templates. For example, DNA template AgNCs (DNA-AgNCs) can display bright light tunable emission colors and enhance stability by adjusting the sequence of the DNA template. Therefore, DNA-AgNCs are promising sensors for DNA.

**Figure 2:**
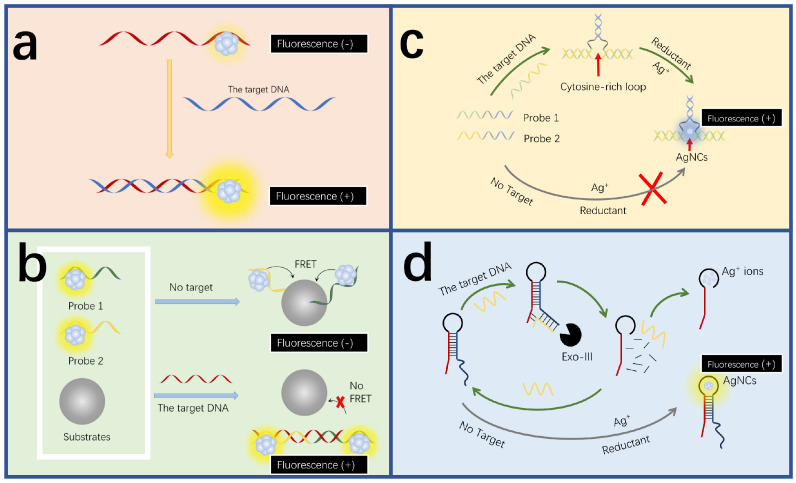
DNA detection mechanisms of fluorescent AgNC-based sensors. (**a**) Direct hybridization-induced fluorescence quenching. The target hybridizes with the DNA-AgNCs to directly change the fluorescence intensity. Redrawn from [89]. (**b**) Indirect hybridization-induced fluorescence quenching. The target hybridizes with the DNA-AgNCs to indirectly change the fluorescence intensity, for example, by disrupting the resonance energy transfer process. Redrawn from [91]. (**c**) The fluorescence quenching by the interaction between sequence bases and AgNCs. The target induces the formation of cytosine-rich structures, favoring the generation of fluorescent AgNCs and thereby increasing the fluorescence intensity. Redrawn from [92]. (**d**) Amplification strategy-assisted DNA detection. The schematic diagram of the label-free assay for enzymatic amplification DNA detection. Redrawn from [93].

High fluorescence performance is critical for accurate DNA detection. Compared with optical quantum dots-QDs and organic dyes, DNA-AgNCs have the advantages of a long fluorescence period and low toxicity [84,86,87]. DNA-AgNCs typically show high sensitivity in DNA detection, allowing AgNC-based sensors to accurately detect DNA without the need for amplification methods. DNA hybridization technology utilizes the complementary pairing between DNA strands to achieve specific recognition of target DNA. The direct hybridization of DNA templates enables highly selective sensing of targets, leading to the development of many sensing platforms [88] (Figure 2a). For instance, Lee et al. discovered that guanine-rich-DNA-AgNCs show highly selective responses to FOXP3 DNA [89], with a limit detection reaching as low as 0.625 μM. The hybridization of AgNCs with a guanine-rich chain and target DNA will produce a three-way target sequence, which will shift the fluorescence spectrum and increase the fluorescence intensity. Changes in fluorescence intensity allow for the quantitative detection of FOXP3 DNA. Zhou et al. used A_20_-C55 DNA-AgNCs as the fluorescence probes of the ratio fluorescence sensor [90]. The probe has good sensitivity, versatility, a high signal-to-noise ratio, etc. When there is no target DNA, it presents a single yellow fluorescence emission at 570 nm. When it hybridizes with target DNA and approaches non-fluorescent auxiliary AgNCs, the yellow fluorescence emission signal decreases, while a new red emission signal increases. In this way, a controllable fluorescence color transition from yellow to red is achieved, offering a suitable platform for analyzing HBV and HIV.

In addition to the direct hybridization strategy, indirect hybridization is another common approach for DNA detection using DNA-AgNCs (Figure 2b). There is also a fluorescence detection strategy that utilizes fluorescence resonance energy transfer (FRET) between dyes and nanomaterials to analyze the object [94,95]. A sensing platform based on carbon nanoparticle (CNP) oxide and DNA functionalization of AgNCs was prepared [91]. The fluorescence resonance energy transfer between AgNCs and CNPs oxide was mainly used to detect HIV-DNA. When the target DNA is absent, the two capture probes after functionalization of AgNCs are adsorbed to CNPs oxide, resulting in the emission of AgNCs being quenched. When the target DNA is present, the two capture probes after AgNCs functionalization hybridize with the target HIV-DNA to form dsDNA. Due to the weak adsorption of dsDNA by CNPs oxide, the two capture probes are far away from the CNPs oxide, resulting in AgNCs emitting light. The sensor can be used to detect single base mismatch and has strong specificity.

The interaction between DNA templates and AgNCs can be also utilized for DNA detection (Figure 2c). The intensity of DNA-AgNCs can be affected by the base sequences of the DNA templates [96]. For example, the high affinity of silver ions with cytosine (C) groups can further promote the formation of AgNCs in situ [97,98]. Guanine (G) can enhance the fluorescence intensity of AgNCs [99]. Based on this feature, Zhang and colleagues developed a labeling-free fluorescence sensing platform based on three-segment-branched DNA-AgNCs [92]. When H5N1 virus DNA is present, complementary base pairing occurs between the probe and H5N1 to form a closed cytosine-rich loop that can chelate Ag^+^ to generate AgNCs. The target gene can be qualitatively and quantitatively detected according to the change of fluorescence intensity. The sensor has a wide linear detection range of 500 pM–2 nM. Zou et al. built up a high-sensitivity detection platform using AgNCs for HIV-1 and HIV-2. The platform is based on fluorescence enhancement of guanine-rich sequence and AgNCs dimerization [100]. When the target DNA is missing, the fluorescence signal at one end of the AgNCs is enhanced due to the presence of a G-rich sequence, while the signal at the other end is enhanced due to the interaction between the two AgNCs. The hybridization of the target DNA with the probe separated the G-rich sequence from the AgNCs. At the same time, it depolymerized the dimerized AgNCs. The fluorescence intensities of the AgNCs at both ends were weakened. This multi-channel analysis platform for the target DNA exhibits high selectivity and a low detection limit of 11 pM.

Early detection of DNA requires sensors to be ultrasensitive. DNA amplification strategies can significantly enhance the sensitivity of sensors by amplifying DNA signals. To date, major multiple amplification strategies for DNA detection include enzyme-free and nuclease-based strategies [101,102,103]. A typical example of enzyme-free amplification is the hybridization chain reaction (HCR) strategy. HCR is an isothermal and enzyme-free process in which the cascade reaction is initiated by the target analyte [88,104,105]. Due to its advantages, such as the autonomous isothermal replication process, high enzyme-free amplification efficiency and controllable kinetics, biosensors based on HCR for DNA detection have been reported [106,107]. Wong and colleagues report a DNA biosensor based on HCR and AgNCs with a detection limit of 3.35 fM [108]. In the absence of DNA-214, the hairpin structures (H1 and H2) do not trigger HCR, and the addition of P1 (the sequence region rich in cytosine) can form a relatively stable P1-H1 double-stranded structure, in which the AgNCs on P2 (the core sequences) remain non-fluorescent. When the target DNA is present, H1 and H2 participate in the amplification process of the HCR, which causes the hybridization of P1 and P2 and generates the emission of red fluorescence. This is because the P1-P2-AgNCs complex promotes the end-to-end transfer of non-fluorescent AgNCs from the P2 end to the cytosine-rich region of P1 and then activates the red-emitting substances. The intensity of this red fluorescence increases as the concentration of the target DNA rises. Chen et al. used the formation of cytosine-rich sequences to promote the in situ generation of AgNCs and developed an autocatalytic strand displacement amplification with continuous HCR for effective coupling of DNA biosensors [109]. In the presence of target DNA, this strategy causes displacement amplification of the autocatalytic chain, generating many identical auxiliary DNA strands (ASs) corresponding to the target DNA within the DNA fragments. These can be used for further hybrid identification and amplification processes. The ASs from the ASDA chain can be used as primers, and they can propagate the HCR between two hairpins (H1 and H2), forming a series rich in cytosine that can promote the in situ generation of AgNCs. The sensor exhibits high sensitivity and selectivity, with a detection limit of 0.16 fM.

Signal amplification based on nuclease includes stand displacement amplification and Exonuclease-III(Exo-III)-assisted amplification (Figure 2d). Exo-III-assisted strategies, in which Exo-III is a sequence-independent enzyme that catalyzes the gradual removal of 3’ hydroxy-terminal single nucleotides [110,111,112], provide a universal platform for DNA detection. The strand displacement amplification method involves polymerase extension and endonuclide cleavage, offering an amplification efficiency up to 10^7^. The strategy is characterized by its homogeneity and label-free nature [113]. Yang and colleagues successfully constructed a fluorescent biosensor platform based on a probe for spherical recognition and a AgNC-based switch initiated by the strand displacement reaction [114]. The sensor has a detection limit of 250 pM, with a good recovery ability. When the target DNA associated with Alzheimer’s disease is present, the capture probe on the surface of the magnetic bead hybridizes with it, forming a blunt 3’ end that further activates the Exo-III enzyme, resulting in the release of the target DNA and the trigger chain. The trigger chain leads to the chain shift reaction, sharply decreasing the fluorescence intensity of AgNCs. While the absence of the target will neither activate the Exo III enzyme nor release the trigger chain, intense red fluorescence can be observed as a result. Shen et al. developed a label-free and visual probe for DNA detection using the hairpin-templated AgNCs [93]. When the target DNA is absent, the hairpin DNA probe leads to the in situ formation of highly fluorescent AgNCs. When the probe recognizes the target DNA, it forms a three-way junction structure. Exo-III gradually degrades the three-way junction structure, destroying the hairpin DNA probe and reducing the amount of highly fluorescent AgNCs. Therefore, the fluorescence intensity is inversely proportional to the target concentration.

The silver nanocluster beacons (AgNCBs) are label-free, activated fluorescent probes with a core consisting of few-atom AgNCs [115]. AgNCBs offer various modalities of responses, including “turn-on” and “chameleon” mechanisms, and they do not rely on FRET for their activation or color change [116]. In comparison to AgNCs, AgNCBs show advantages in target recognition, ease in activation, and exhibit greater resistance to enzymatic degradation [117].

Most reported AgNCB-based sensors employ a single-emission on/off signal readout strategy, which is limited by sensing interference from environmental and/or experimental conditions. However, the proportional fluorescence biosensor based on AgNCBs can simultaneously record the relative fluorescence changes of two well-separated wavelengths, which can further improve the sensitivity and accuracy of biosensing by eliminating systematic errors and can more accurately quantify the target concentration [118,119,120]. The “chameleon” in NCBs shows that when the converter is close to DNA-AgNCs, a more resolved multicolor fluorescence signal can be obtained, usually using the ratio of fluorescence intensity as the output signal.

**Figure 3:**
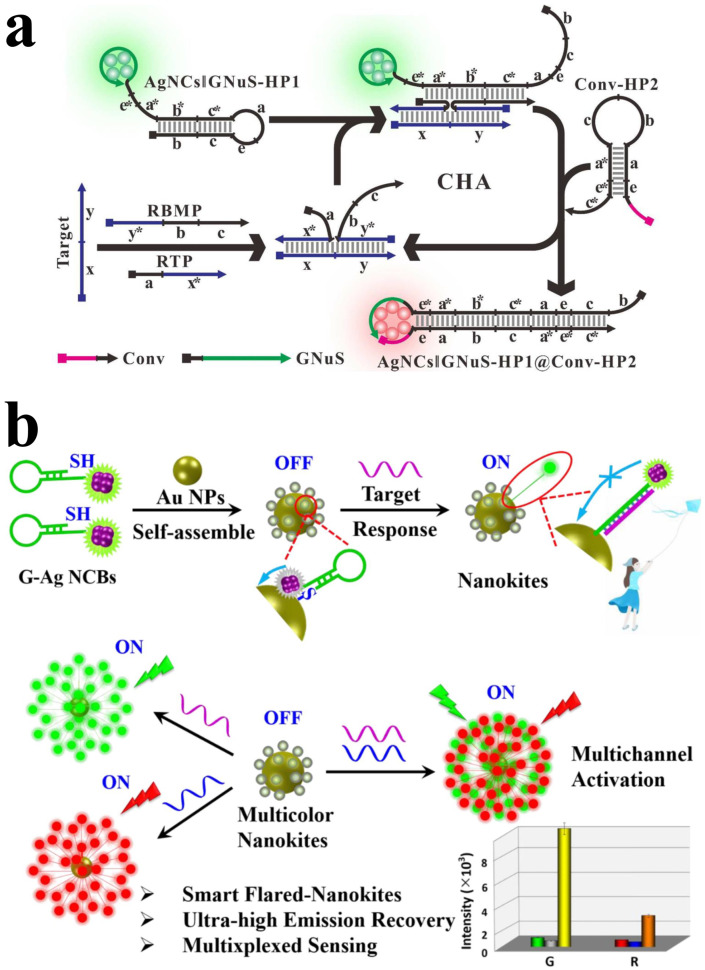
(**a**) The schematic of ratio-fluorescent biosensing platform based on RCA-amplified AgNCBs. Reproduced with permission from [118]. Copyright 2017 American Chemical Society. (**b**) Schematic illustration of dual DNA sensors based on AgNCBs and AuNPs. Reproduced with permission from [117]. Copyright 2023 Elsevier.

AgNCBs are commonly used as a fluorescence ratiometric sensor, detecting DNA by fluorescence emission wavelength more than intensity. The fluorescence detection mechanism based on AgNCB is unique to AgNCs. In Ge et al.’s study, a fluorescence ratio biosensing platform was developed for detecting Werner syndrome (WS)-related DNA using a catalytic hairpin assembly expansion strategy based on AgNCBs [118]. Ratiometric catalyzed-assembly AgNCBs can detect target DNA by converting green-emitting AgNCs into red AgNCs when they are near a particular DNA fragment (Figure 3a). With a detection limit of 8.5 pM and a wide linear detection range, the sensor is suitable for detecting WS -related gene targets in human serum. Ge et al. developed a DNA fluorescence ratio sensor fort Hepatitis-A (HAV) based on AgNCBs [119]. In this sensor, the fluorescence color transition from red to green indicates whether the specific DNA fragment is linked to the green nucleation sequence (GNuS) of AgNCs. The presence of target DNA disrupts the hairpin structure of NCBs, physically separating the special DNA fragment from GNuS and resulting in a green emission. While in the absence of target DNA, the hairpin structure of NCBs remains intact, keeping the converter and GNuS connected and generating a red emission.

AgNCBs can also be used for dual DNA detection, which can avoid false diagnoses and overcome the time-consuming drawback observed in traditional methods [121]. Feng et al. developed a sensing platform for the simultaneous detection of H1N1 and HIN5 DNA of influenza viruses (Figure 3b), which is capable of identifying subtype genes within the same virus [117]. The sensor is constructed using AgNCBs and AuNPs, where there are two types of AgNCBs responding to two different DNA targets, realizing a dual detection. When there is no target, the fluorescence is quenched by the energy transfer between AuNPs and AuNCs. In the presence of the target DNA, the hairpin-structure AgNCBs transform into rigid double-stranded (dsDNA), separating AuNPs and AuNCs, disrupting the energy transfer and increasing the fluorescence intensity. Therefore, the notable fluorescence signals reflect the presence of DNA targets.

Currently, AgNCB-based sensors are not only used for detecting multiple DNA targets from viruses but also for sensing multiple analytes, including DNA, small molecules and proteins. Liu et al. successfully synthesized activated AgNCBs using a well-designed multifunctional single-stranded DNA sequence [122], which comprises a segment capable of forming AgNCs, a target recognition segment, and a segment used for attaching quenching agents. The sensor works on the FRET mechanism, where the interaction with the target molecule disrupts the beacon’s hairpin structure, leading to detachment from the quencher and a consequent rise in fluorescence. By varying the design of the target recognition sequence, this sensor facilitates the detection of multiple DNA sequences, small molecules, and proteins.

In this chapter, DNA detection sensors based on AgNCs are categorized into two major groups: those based on AgNCs and those based on AgNCBs. The data presented above indicate that silver ions exhibit a strong affinity for cytosine, and DNA sequences rich in cytosine significantly facilitate the formation of AgNCs. Reactions such as strand displacement, HCR and Exo-III-assisted amplification are employed to amplify the detection signal, thereby achieving a low detection limit. Table 2 reveals that the minimum detection limit can reach the pM level, exhibiting a high sensitivity.

### 3.2. AuNCs for DNA Detection

AuNCs emerge as increasingly popular DNA sensors [139,140], due to their superior biocompatibility and stability [141,142,143,144,145]. DNA and protein-templated AuNCs stand out as the most commonly used ones for DNA detection, among all the AuNCs capped by different ligands, like thiols [146], polymers [147], DNA [148], peptides [149], and proteins [150].

**Figure 4:**
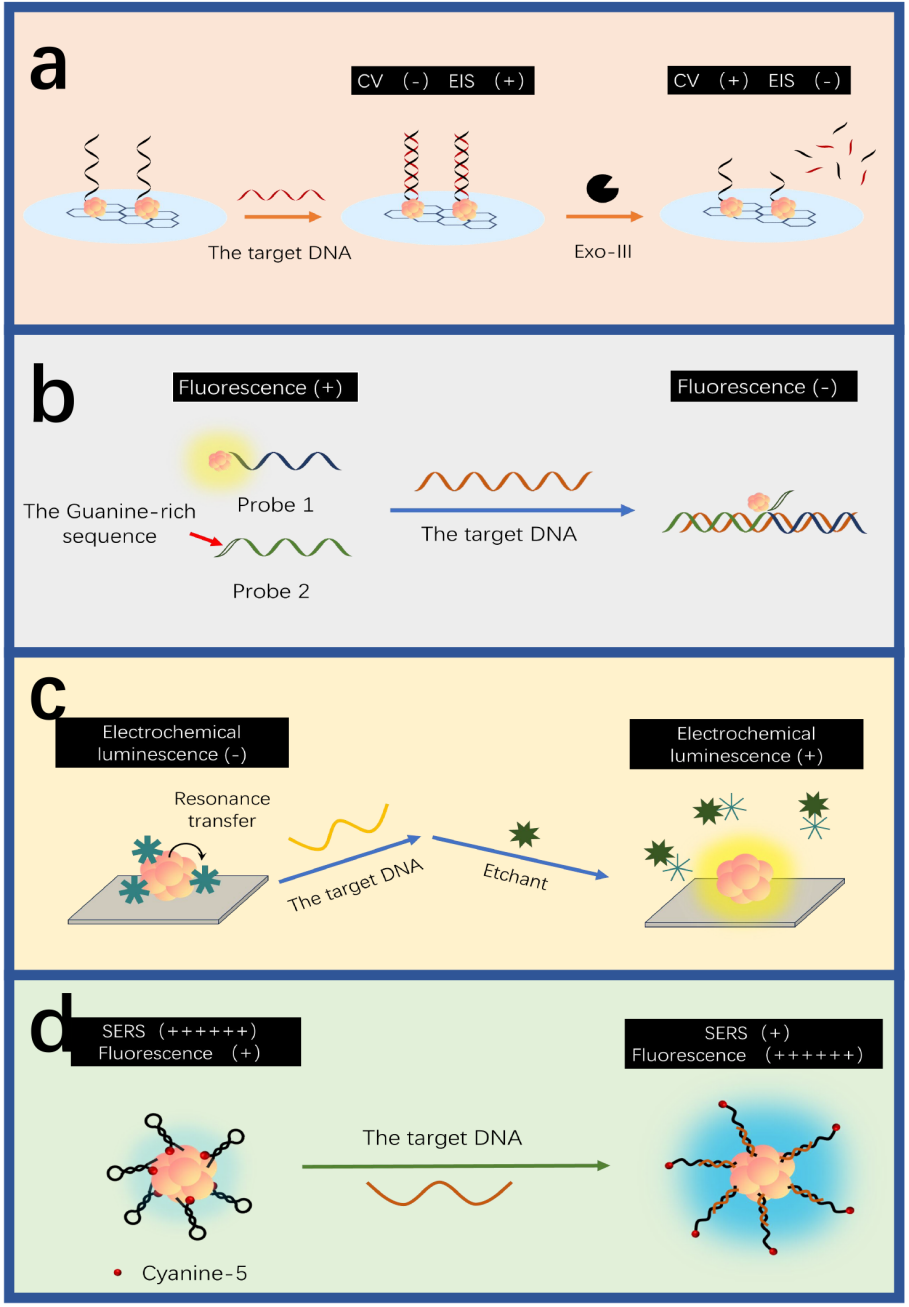
DNA detection mechanisms of AuNC-based sensors. (**a**) The electrochemical detection using Exo III-assisted target cycle amplification strategy. The hybridization between the target and DNA-AuNCs decreases the impedance. Redrawn from [124]. (**b**) The fluorescent detection. The hybridization among the target, the guanine-rich sequence and the DNA-AuNCs decrease the fluorescence intensity. Redrawn from [127]. (**c**) Electrochemical-luminescent detection. The target disrupts the resonance transfer between AuNCs and MnO_2_, increasing the intensity of electrochemical luminescence. Redrawn from [128]. (**d**) The detection based on SERS. The target hybridizes with the hairpin DNA-AuNCs to yield strengthened SERS signals. Redrawn from [130].

Electrochemical sensing is widely used in DNA detection for its advantages of high sensitivity, fast response and low cost [151,152,153,154] (Figure 4a). Shamsipur et al. developed an electrochemical sensor for BCR/ABL fusion genes based on graphenes covered by hemoglobin-templated AuNCs [123]. When the complementary DNA hybridizes with the probe ssDNA, methylene blue in the probe ssDNA is released upon interaction with the complementary DNA, reducing the differential voltammetry current and increasing the electrochemical impedance spectroscopy resistance. The detection limit of the sensor is 0.037 fM. Wang and colleagues pioneered the integration of AuNCs/graphene (GR) nanocomposites with an Exo-III-mediated target recycling strategy to fabricate an electrochemical DNA sensor [124]. The target DNA initiates the Exo-III cascade target cycle process, producing cleavage products that facilitate the connection between the capture probe and AuNCs/GR-DNA-alkaline phosphatase, which is anchored on the electrode. AuNCs/GR-DNA-alkaline phosphatase further catalyzes the deposition of silver to form AgNPs on the electrode, generating electrical signals that are used for the quantitative DNA detection, with a detection limit of 0.057 fM. Wang et al. developed an HIV-DNA electrochemical biosensor based on graphene-stabilized AuNCs (GR/AuNCs) [125]. The glassy carbon electrode modified by GR/AuNCs provides more fixed sites for the capture probe. The target DNA hybridizes with the capture probe to form a double-stranded DNA structure. This triggers the Exo-III enzyme to cleave the 3’ end of the capture probe, releasing methylene blue molecules from the electrode surface and recovering the target DNA. This results in current changes, allowing for the quantitative detection of HIV-DNA, with a detection limit reaching 30 aM.

**Figure 5:**
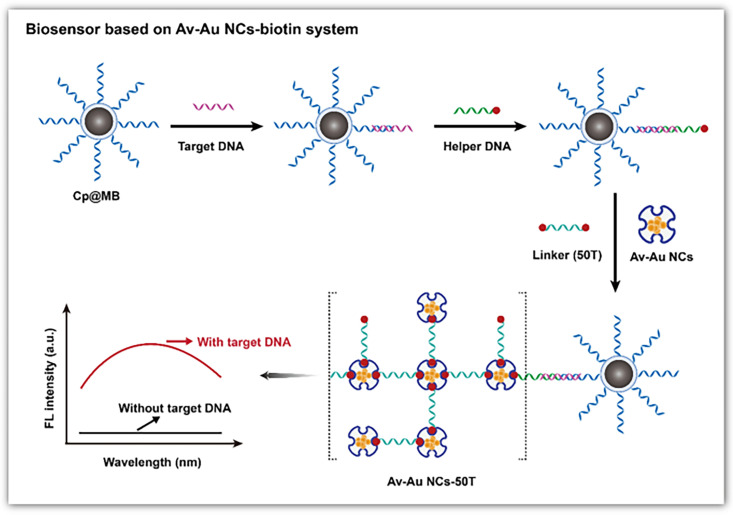
Schematic illustration of the fluorescent DNA biosensor based on the Av-AuNCs-biotin signal amplification system. Reproduced with permission from [126]. Copyright 2022 Springer.

AuNCs are known for their superior fluorescence properties, which can be utilized for sensitive, selective and quantitative DNA detection (Figure 4b). Tang et al. used avidin-stabilized AuNCs (Av-AuNCs) as a fluorescence sensor for DNA [126] (Figure 5). When the target DNA exists, it hybridizes with the capture DNA chain and the helper DNA, forming a sandwich structure. Subsequently, a linker chain modified with biotin at both ends is added to crosslink Av-AuNCs and magnetic beads, resulting in strong fluorescence signals. Thus, the fluorescence intensity rises with the target concentration, and the linear detection range is 20 μM–0.2 nM. Unlike the fluorescence quenching mechanism of AgNCs based on Förster resonance energy transfer, the fluorescence quenching of AuNCs is mainly based on the mechanism of photoinduced electron transfer. Wang et al. developed a marker-free sensor for H1N1 DNA based on proximity-dependent hybridization and guanine-induced fluorescence quenching [127]. This fluorescent sensing platform has a detection limit of 200 pM. Upon hybridization of the target DNA with P2DNA—which includes a template sequence for the synthesis of AuNCs and hybridization sequences—and 6G probe 2—which comprises guanine-rich overhang sequences and hybridization sequences—a “Y”-shaped structure is formed. This configuration leads to substantial fluorescence quenching when in proximity to DNA-AuNCs and G-rich sequences, likely due to the photoinduced electron transfer between the AuNCs and the guanine-rich DNA. When there is no target, the 6G probe 2 emits strong fluorescence at 475 nm of excitation. The target concentration is inversely proportional to the fluorescence intensity.

In addition to electrochemical and fluorescent methods, electrochemical luminescence (ECL) is also considered as a powerful and promising technique for DNA detection due to its rapid responses, low background noises, and high sensitivity [155,156,157,158] (Figure 4c). Hong et al. designed an ECL sensor based on AuNCs to probe HPV-16 DNA, which has a detection limit of 6.8 aM [128]. In the absence of the target DNA, the ECL signal is weak due to the resonant transfer between AuNCs and manganese dioxide particles. However, when the target DNA is present, it hybridizes with the capture probe and the biotin probe to form a “sandwich” structure known as the biotinylated DNA complex. This triggers a reaction between streptin-alkaline phosphatase and the biotinylated DNA complex to produce ascorbic acid, which etches manganese dioxide particles to curb the resonant transfer between manganese dioxide particles and AuNCs, resulting in significant ECL signals. The concentration of the target DNA is thereby proportional to the intensity of ECL signals. Liu and colleagues developed an ECL biosensor using Clustered Regularly Interspaced Short Palindromic Repeats (CRISPR)-associated 12a for target amplification in the HIV-DNA testing [129]. Archaea and bacteria can be used for signal amplification in the CRISPR/Cas system, which is widely used for the accurate diagnosis of nucleic acids [159,160]. The CRISPR/Cas system has a biological activity known as “genetic scissors”, which can identify and cut the specific DNA target sequences upon the guidance of CRISPR RNA, further activating its cutting ability [161,162,163,164]. The sensor employed L-methionine-stabilized AuNCs (Met-AuNCs) as an efficient ECL emitter. Ferrocene-tagged thiolated single-strand DNA (SH-ssDNA-FC) was introduced to quench the Met-AuNCs launch, leading to weak ECL signals. When the target DNA was present, the nonspecific ssDNA trans-cutting ability of Cas12a was activated, resulting in the indiscriminate cutting of SH-ssDNA-FC. As a result, ECL signals were greatly recovered. The target DNA can be quantitatively determined by correlating ECL signals with different target concentrations.

Surface-enhanced Raman scattering (SERS) can sensitively identify patterns of biomarkers in a single spectrum without the need for complex array structures of sensors [165,166,167,168,169,170]. Guo et al. used asymmetric polymerase chain reaction (PCR) and SERS to detect EGFR mutations [130]. In the absence of the target DNA, when the molecular beacon was in a loop-stem structure, the SERS signals of AuNCs were strong. However, when the target was present, multiple target objects produced by PCR could hybridize with the molecular beacon, forming a double-helix structure and keeping 5’ Cy3 far away from AuNCs. This significantly reduces the SERS signal.

This part focuses on AuNC-based sensors that can detect DNA through various modalities, including electrochemical, fluorescent, ECL, and SERS methods. Most of these sensors utilize AuNCs template by DNA and proteins, with those integrated with CRISPR/Cas systems showing significantly improved sensitivity in detection. As shown in Table 1, the minimum detection limit can reach the aM level.

### 3.3. CuNCs for DNA Detection

DNA-templated CuNCs (DNA-CuNCs) emerge as promising DNA sensors with ease in preparation and relatively low cost [171,172], despite them not been as widely reported as AgNCs and AuNC-based sensors [75].

CuNCs can show better performance in DNA detection than AgNCs and AuNCs. Tao et al. developed a fluorescent sensor based on CRISPR-Cas12a enzyme and MNCs, including AuNCs, AgNCs and CuNCs, for the detection of hepatitis B virus (HBV)-DNA [133]. The presence of the target causes Cas12a to indiscriminately cut the DNA probe, inhibiting the formation of MNCs and thus decreasing the fluorescence intensity, which is inversely proportional to the target concentration. Compared with AuNCs and AgNCs, CuNCs show faster responses and higher sensitivity, reaching a response time of 25 min and a detection limit as low as 0.54 pM.

**Figure 6:**
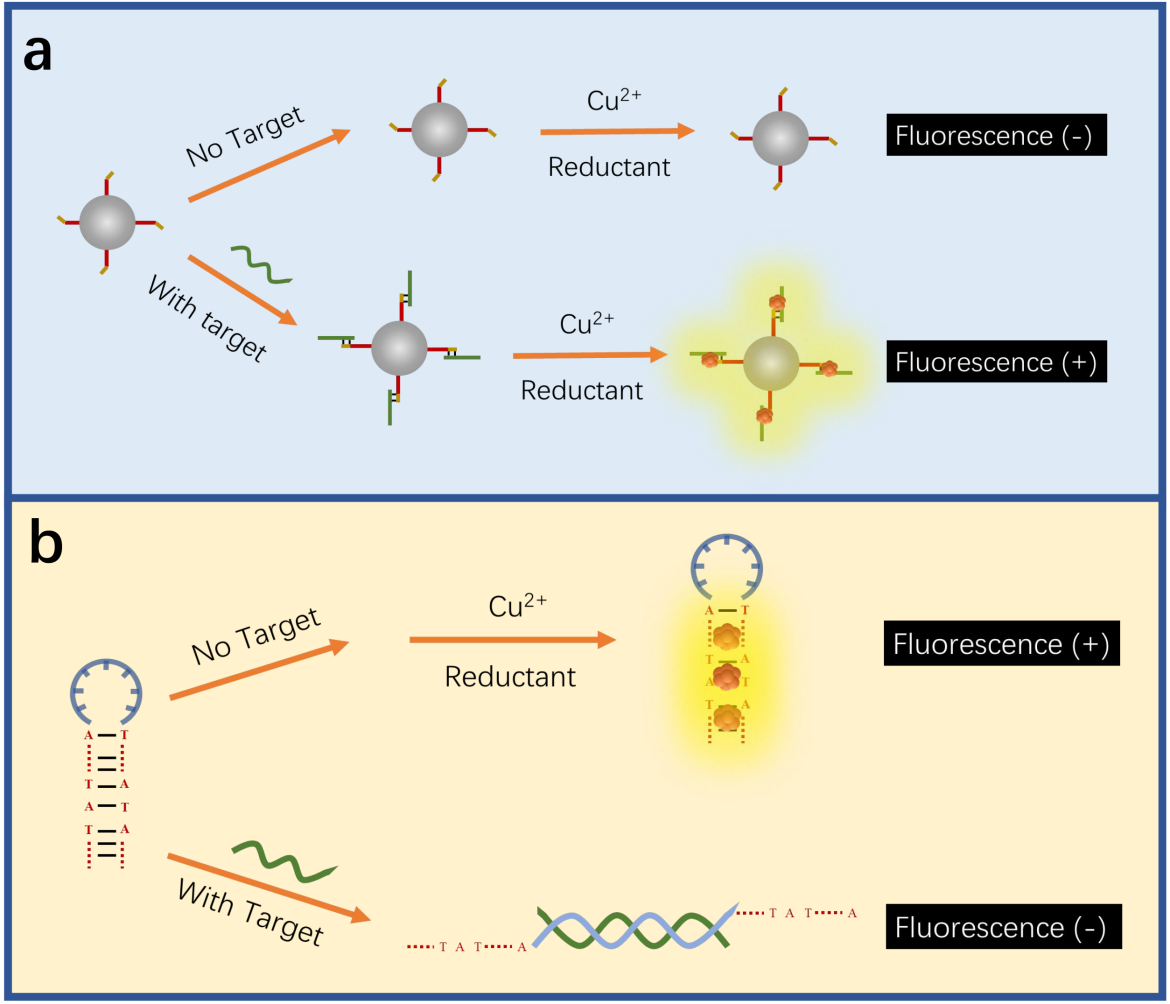
Schematic illustration of CuNC-based sensors. (**a**) The fluorescent detection based on the in situ formation of fluorescent CuNCs. The presence of a target produces favorable structures for generating fluorescent CuNCs, increasing the fluorescent intensity. Redrawn from [132]. (**b**) The fluorescent detection based on the non-formation of fluorescent CuNCs. The target destroys the hairpin structure, which favors the formation of fluorescent CuNCs, decreasing the fluorescent intensity. Redrawn from [134].

**Figure 7:**
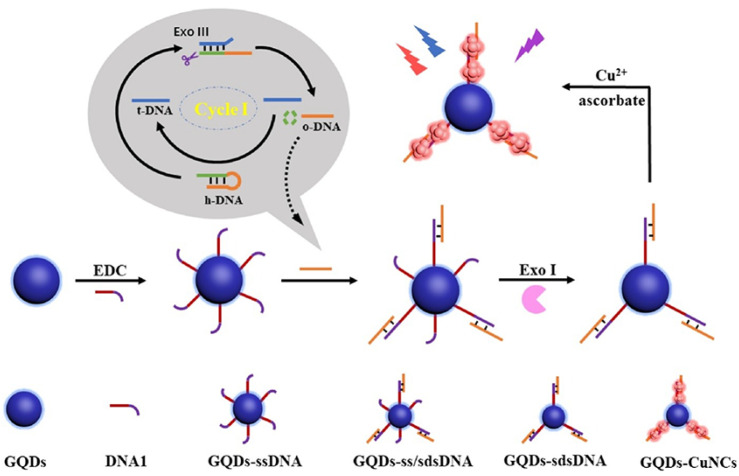
Schematic illustration of ratio fluorescence detection of HTLV-1 DNA based on the in situ generation of CuNCs on the DNA modified GQDs. Reproduced with permission from [132]. Copyright 2021 Elsevier.

Different from AgNCs and AuNCs, the fluorescence detection of CuNCs is mainly based on the interaction between DNA bases and structures and CuNCs to achieve the detection of target DNA. The adenine and thymine-rich sequences serve as an effective template for highly fluorescent CuNCs [173,174] (Figure 6a). This feature can be utilized in indirect probing of DNA, where the target concentration is determined by the change in fluorescence intensity induced by the formation of CuNCs in adenine and thymine-rich DNA. For example, Chen et al. utilized this feature to construct a fluorescent sensor for lymphotropic virus DNA [132] (Figure 7). The sensor has a detection range of 20 pM–12 nM. When the target DNA is present, an amplification strategy using the EXO-III-assisted target recycling is adopted to produce a large amount of AT-rich single DNA(oDNA). The DNA1 on the graphene quantum dots (GQDs) can then hybridize with the oDNA to form GQDs-ss\sdsDNA, which is resistant to digestion because of the EXO-III. On the GQDs-sdsDNA, fluorescent CuNCs can be generated. The change in fluorescence intensity reveals the concentration of the target DNA. Similarly, Chen et al. developed a fluorescence sensor for viral genetic DNA based on the in situ formation of fluorescent CuNCs from AT-rich double-stranded DNA [131]. The sensor uses target recycling amplification and magnetic separation strategies, with a detection limit as low as 1 pM. When the target DNA is present, a large number of DNA output regions (oDNA) are released after amplification of the target cycle. oDNA cannot only hybridize with the capture DNA, which is coupled on the surface of magnetic beads, but also with the AT-rich dsDNA in the solution, which is used for the formation of fluorescent CuNCs. The remaining dsDNA in the solution can be obtained by magnetic separation and used to form CuNCs. By correlating the fluorescent intensity of the as-formed CuNCs with the concentration of remaining dsDNA, the concentration of the target DNA can be thereby determined.

The hairpin structures of the adenine and thymine-rich DNA are more efficient in producing highly fluorescent CuNCs than other DNA structures [175] (Figure 6b). Moreover, this feature can also be utilized to probe DNA. Normally, the DNA hybridization between probes and analytes disrupts the hairpin structure, which is rich in adenine and thymine bases, causing a decrease in fluorescence intensity due to the disruption of CuNCs formation. For instance, Liu and others built up a qualitative and quantitative sensor for *chlamydia trachomatis* OMP1 gene, in which the target DNA can disrupt the hairpin structure of the probe for generating fluorescent CuNCs, leading to a decrease in fluorescence intensity [134]. The concentration of the target DNA is linearly correlated with the fluorescence intensity, and the detection limit is 18.5 nM. Liu and others reported a fluorescent sensor for the breast cancer 1 (BRCA1) gene, with a detection limit of 2 nM, and a good recovery rate in bovine serum [135]. The target DNA hybridizes with the ring region of the hairpin probe, reducing the fluorescence intensity as a result of less formation of highly fluorescent CuNCs within the hairpin structure. The fluorescence intensity decreases with the increasing concentration of the BRCA1 gene.

A lower formation of CuNCs within DNA structures leads to a lower fluorescent intensity, which can be either caused by fewer as-formed highly fluorescent CuNCs, as indicated in the above-mentioned work, or more fluorescence quenchers, such as Cu^2+^ ions. Singh and others constructed a fluorescent sensor for DNA damage, utilizing Cu^2+^ ions [176], which are produced by the inhibition of CuNC formation, as the fluorescence quencher. Basic sites are present in the damaged part of DNA, hindering the generation of CuNCs within the structure and increasing the amount of Cu^2+^ ions in the solution. Cu^2+^ ions are then detected by fluorescent carbon dots, whose fluorescent intensity is proportionally quenched, quantitatively reflecting the presence of DNA damage.

In summary, adenine- and thymine-rich sequences and their hairpin structures favor the formation of fluorescent CuNCs, which can be utilized for CuNC-based sensors to detect DNA. Notably, the detection limit of CuNC-based DNA sensors can be as low as 0.54 pM, which is higher than those of their AgNC and AuNC counterparts. Compared with AuNCs and AgNCs, there are fewer reports on CuNC-based sensors for DNA detection, due to the intrinsically high difficulty in obtaining ultra-small CuNCs.

### 3.4. Alloy MNCs for DNA Detection

Alloy MNCs can show superior electronic and optical properties because of their multi-metal composition [177]. For example, bimetallic Au/AgNCs have a higher fluorescence quantum yield and better stability compared to AuNCs or AgNCs [178]. Multi-recognition molecular linking and the integration of multiple signals can optimize alloy nanoclusters (such as AuIAgNCS) for double or multi-target DNA detection. Bimetallic Cu/AuNCs also show enhanced fluorescence and a long fluorescent lifetime of 6.55 μs compared to AuNCs [179]. The enhanced performance makes alloy MNCs promising, sensitive and stable sensors for DNA detection.

Dehghani and others developed a rapid sensor for jejunum-bending bacteria DNA based on Au/AgNCs [136]. DNA detection is realized by hybridizing the DNA probe with the target DNA, which results in the fluorescence quenching of Au/AgNCs. The linear detection range is 10 to 30 pM, with a detection limit of 4.4 pM.

**Figure 8:**
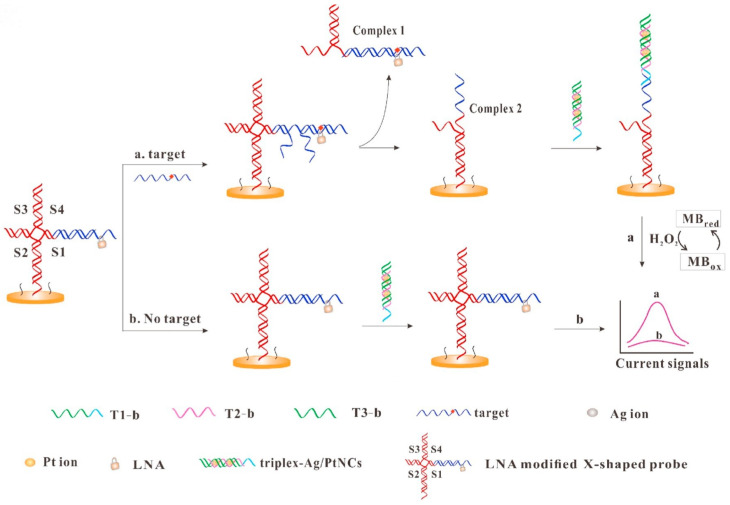
Schematic illustration of electrochemical detection of DNA based on the triplex-Ag/PtNCs and LNA-modified X-shaped DNA probe. Reproduced with permission from [137]. Copyright 2020 Elsevier.

Compared with AgNCs, Ag/PtNCs show better homogeneity and peroxisase-like activity. Wu and others used DNA-templated Ag/PtNCs as an electrochemical probe, integrating it with a locked nucleic acid-modified X-shape probe for the identification of single nucleotide polymorphisms (Figure 8) [137]. The presence of target DNA triggers the strand displacement reaction between the target DNA and the X-type probe, leading to the division of the X-type probe to form Complex 1 and Complex 2. Complex 2, which remains bound to the electrode surface, can hybridize with the highly catalytic triplex-Ag/PtNCs to further catalyze the oxidation of methylene blue, leading to a substantial increase in electrical signals. The target concentration is proportional to the current, and the sensor has a detection limit of 0.8 fM.

The immediate detection of biomolecules is critical in cancer diagnosis [180], in which alloy NCs can serve as an effective sensor with high sensitivity. Wasfi et al. developed a graphite oxide-based field-effect transistor (FET) for real-time DNA detection, whose graphite oxide channel is modified by trimetal nanoclusters consisting of gold, silver and platinum, achieving a detection limit of 1.28 nM [138]. The presence of target DNA produces more holes in the channel to trap electrons, thereby increasing resistance and reducing current. The sensor’s current decreases with the rising concentration of target DNA.

## 4. Conclusions

MNCs have attracted significant attention in biosensors due to their high photoluminescence performance, redox capabilities, and unique electronic structures. Currently, there are many synthetic methods to obtain MNCs. Various DNA amplification strategies, including the hybridization chain reaction strategy, the catalytic chain moving amplification and the target cycle amplification, have significantly enhanced the sensitivity of sensors. Notably, the new CRISPR/Cas system is particularly useful in increasing detection limits. In recent decades, there has been rapid development in the biomedical detection of DNA with optical and electrochemical sensors based on MNCs. These sensors enable the early detection of DNA-related diseases, such as cancers and tumor metastasis, although some of them can be only used under certain clinical conditions. The stability of MNC-based sensors still needs improvement, which presents a great challenge. The stability of MNCs can be improved by selecting appropriate ligands and applying proper doping methods. The use of appropriate encapsulation materials for packaging biosensors and application of protective coatings can improve the stability and biocompatibility of MNC-based biosensors during long-term applications. However, alloy MNCs remain under-studied. Based on MNCs, sensors will play an increasingly important role in DNA detection. Moreover, there is a need to reduce interference factors in complex samples, overcoming the challenges of sensor integration and miniaturization. This is of great significance for the development and application of MNC-based sensors in the early detection of clinical diseases. Overall, biosensors based on MNCs have diverse detection capabilities. Besides being able to conduct precise detection in the early stage of DNA infection, they can also effectively monitor viral RNA. At the same time, they are capable of carrying out highly sensitive detection of cancer-related miRNA and epigenetic modifications, providing more comprehensive and in-depth technical support for the diagnosis and research in the biomedical field.

## Figures and Tables

**Table 1:** Comparison of the yield, reproducibility and scalability of different synthesis methods for MNCs.

Synthesis Method of MNCs	Yield	Scalability	Reproducibility
Chemical reduction	Regulated by reactant concentration, reducing agent, temperature, and time	Higher	Higher
Ligand exchange	Governed by equilibrium constant, ligand concentration, and reaction activity	Low	Low
Chemical etching	Depends on material surface area, etchant concentration, and etching time	Medium	Medium
Microwave-assisted and green synthesis techniques	Influenced by microwave power, radiation time, reactant concentration, green solvents, and biocatalysts	High	High

**Table 2:** Recently reported MNCs for DNA detection.

Material	Synthesis Template	Detecting Technology	Target	Detection Limit (M)	Linear Range (M)	Ref.
AgNCs	DNA	Fluorescence	FOXP3-DNA	$6.25\times 10^{-7}$	-	[89]
AgNCs	DNA	Ratiometric fluorescence	HIV-DNA	$5\times 10^{-9}$	$5\times 10^{-9}$–$10\times 10^{-7}$	[90]
			HBV-DNA			
AgNCs	-	Fluorescence	HIV-DNA	$4\times 10^{-8}$	$1\times 10^{-9}$–$5\times 10^{-8}$	[91]
AgNCs	-	Fluorescence	H5N1-DNA	$5\times 10^{-10}$	$5\times 10^{-10}$–$2\times 10^{-9}$	[92]
AgNCs	DNA	Fluorescence	two HIV DNA	$1.2\times 10^{-11}$	$2\times 10^{-8}$–$7\times 10^{-8}$	[100]
AgNCs	DNA	Fluorescence	DNA-214	$3.35\times 10^{-15}$	0–$1\times 10^{-9}$	[108]
AgNCs	DNA	Electrochemistry	-	$1.6\times 10^{-16}$	$1\times 10^{-11}$–$2.5\times 10^{-8}$	[109]
AgNCs	DNA	Fluorescence	Alzheimer disease DNA	$2.5\times 10^{-10}$	$2.5\times 10^{-10}$–$2.5\times 10^{-8}$	[114]
AgNCs	DNA	Fluorescence	-	$2.8\times 10^{-9}$	$5\times 10^{-9}$–$5\times 10^{-8}$	[93]
AgNCBs	DNA	Fluorescence	H1N1-DNA	$1\times 10^{-11}$	$1\times 10^{-12}$–$2\times 10^{-10}$	[117]
			H5N1-DNA			
AgNCBs	DNA	Ratiometric fluorescence	WS-DNA	$8.5\times 10^{-12}$	0–$8\times 10^{-8}$	[118]
AgNCBs	DNA	Ratiometric fluorescence	HAV-DNA	$5\times 10^{-10}$	$1\times 10^{-9}$–$5\times 10^{-7}$	[119]
AuNCs	hemoglobin	Electrochemistry	BCR/ABL-DNA	$3.7\times 10^{-17}$	$1\times 10^{-14}$–$1\times 10^{-12}$	[123]
AuNCs	graphene	Electrochemistry	-	$5.7\times 10^{-17}$	$2\times 10^{-14}$–$2\times 10^{-12}$	[124]
AuNCs	graphene	Electrochemistry	HIV-DNA	$3\times 10^{-17}$	$1\times 10^{-16}$–$1\times 10^{-14}$	[125]
AuNCs	avidin	Fluorescence	-	$4.3\times 10^{-11}$	$2\times 10^{-10}$–$2\times 10^{-8}$	[126]
AuNCs	DNA	Fluorescence	H1N1-DNA	$2\times 10^{-10}$	$1\times 10^{-10}$–$1\times 10^{-8}$	[127]
AuNCs	-	Electrochemiluminescence	HPV16 E7-DNA	$6.8\times 10^{-18}$	$1\times 10^{-16}$–$1\times 10^{-14}$	[128]
AuNCs	-	Electrochemiluminescence	HPV-16DNA	$4.8\times 10^{-13}$	$1\times 10^{-12}$–$1\times 10^{-10}$	[129]
AuNCs	L-methionine	Surface-enhanced Raman	ctDNA	$4.24\times 10^{-15}$	0–$1\times 10^{-12}$	[130]
		spectroscopy				
CuNCs	DNA	Fluorescence	-	$1\times 10^{-12}$	$5\times 10^{-12}$–$5\times 10^{-11}$	[131]
CuNCs	DNA	Ratiometric fluorescence	HTLV-1	$1\times 10^{-11}$	$2\times 10^{-11}$–$1.2\times 10^{-9}$	[132]
CuNCs	DNA	Fluorescence	HBV-DNA	$5.4\times 10^{-13}$	$5\times 10^{-12}$–$1\times 10^{-10}$	[133]
CuNCs	DNA	Fluorescence	C.trachomatis DNA	$1.85\times 10^{-8}$	$5\times 10^{-8}$–$1\times 9.5^{-7}$	[134]
CuNCs	DNA	Fluorescence	BRCA1-DNA	$2\times 10^{-9}$	$2\times 10^{-9}$–$6\times 10^{-7}$	[135]
Au/AgNCs	DNA	Fluorescence	campylobacter jejuni-DNA	$4.4\times 10^{-12}$	$1\times 10^{-11}$–$3\times 10^{-9}$	[136]
Au/Ag/PtNCs	DNA	Electrochemistry	β-thalassemia-DNA	$8\times 10^{-16}$	$1\times 10^{-15}$–$1\times 10^{-13}$	[137]
Ag/PtNCs	-	Field-effect transistor	-	$1.28\times 10^{-9}$	-	[138]

## Data Availability

Not applicable.
